# Life Course Impact of Glucocorticoids During Pregnancy on Muscle Development and Function

**DOI:** 10.3389/fanim.2021.788930

**Published:** 2021-12-08

**Authors:** Yang Liu, Qiyue Ding, Wei Guo

**Affiliations:** Department of Animal and Dairy Sciences, University of Wisconsin-Madison, Madison, WI, United States

**Keywords:** cardiovascular disease, fetal programming, glucocorticoids, maternal stress, muscle development

## Abstract

Maternal stress, such as maternal obesity, can induce severe gestational disease and hormonal disorder which may disrupt fetal organ maturation and further cause endangered early or future health in offspring. During fetal development, glucocorticoids are essential for the maturation of organ systems. For instance, in clinical applications, glucocorticoids are commonly utilized to pregnant women with the risk of preterm delivery to reduce mortality of the newborns. However, exposure of excessive glucocorticoids at embryonic and fetal developmental stages can cause diseases such as cardiovascular disease and muscle atrophy in adulthood. Effects of excessive glucocorticoids on human health are well-recognized and extensively studied. Nonetheless, effects of these hormones on farm animal growth and development, particularly on prenatal muscle development, and postnatal growth, did not attract much attention until the last decade. Here, we provided a short review of the recent progress relating to the effect of glucocorticoids on prenatal skeletal muscle development and postnatal muscle growth as well as heart muscle development and cardiovascular disease during life course.

## INTRODUCTION

Early life events are critical to growth performance and health throughout the life course of an animal including human beings. Unfavorable maternal environmental changes are associated with many types of abnormalities including cardiovascular disease, obesity, insulin resistance, and metabolic syndrome in offspring later life, which is known as the disease origins of adult disease or developmental programming (Barker et al., 1993; Friedrich, 2002; Boney et al., 2005). Developmental programming is defined that challenges during critical developmental windows cause deterministic consequences in developmental and health trajectory in later life. It is also called fetal programming since it occurs during embryonic and fetal development (Kwon and Kim, 2017). The study of fetal programming can be traced back to around a century ago (Cox et al., 2012), however, the concept was just defined by Dr. Barker about half century ago (Barker and Osmond, 1986). Since then, many studies in humans and animal models support the hypothesis that poor nutrition *in utero*, maternal stress (e.g., depression, anxiety, fatigue, toxic exposure), exogenously administered hormones (e.g., synthetic glucocorticoid, sGC), and other factors could change fetal structure, function, and metabolism, leading to a long-last effects on offspring throughout the whole life. Moreover, metabolic syndrome such as cardiovascular disease in humans and offspring growth performance in livestock are the mostly reported consequences of those adverse factors (Nathanielsz, 2006; Barker, 2007; Gicquel et al., 2008; Beauchamp et al., 2015; Sand et al., 2019; Davies et al., 2021). Therefore, understanding of the underpinning mechanisms of fetal programming could provide cues to develop intervention strategies for poor postnatal growth performance in domestic animals and adult chronic diseases in humans caused by maternal stresses (Cox et al., 2012).

Glucocorticoids are the major stress hormones secreted by the adrenal gland in response to stress, which is regulated by the hypothalamic-pituitary-adrenal (HPA) axis (Gicquel et al., 2008). In the canonical signaling pathway, glucocorticoids function through binding glucocorticoid receptor (GR), which mainly localizes in the cytoplasm of cells and forms a protein complex including hsp90, hsp70, and p23 in the absence of the hormones. After binding with glucocorticoid, GR is dissociated from the complex and its nuclear localization signals are exposed. After GR is transported into nucleus, it regulates gene expression *via* direct binding with the glucocorticoid response element (GRE) of the targeted genes, or interacting with other transcriptional factors such as AP1, NF-κB, and STATs (Oakley and Cidlowski, 2013; Vitellius et al., 2018). Glucocorticoids play a very wide role in postnatal life including the regulation of homeostasis, growth, cell proliferation and differentiation, apoptosis, and metabolism (Fowden and Forhead, 2004; Grad and Picard, 2007; Cain and Cidlowski, 2015). However, glucocorticoids are also critically important in prenatal life in which these hormones are essential for the development and maturation of fetal organ systems such as respiratory system, neural system, endocrine system, gastrointestinal system, renal system, and muscle system (Agnew et al., 2018; Song et al., 2019a). A myriad of studies demonstrated that excessive endogenous glucocorticoids resulting from maternal stress or exogenous glucocorticoids due to antenatal treatment to reduce preterm delivery in pregnancy result in reduced birth weight, fetal growth restriction and adverse effects in adult life such as heart disease, metabolic syndrome, hypertension, and diabetes mellitus (Asztalos, 2012; Carson et al., 2016; Kemp et al., 2016; Agnew et al., 2018). In this short review, we mainly focused on discussing effects of glucocorticoids on striated muscle development and function including skeletal muscle and cardiac muscle. We first discussed the endogenous glucocorticoids synthesis and metabolism in fetuses. Then we discussed effects of glucocorticoids on prenatal skeletal muscle development and postnatal muscle growth as well as prenatal heart muscle development and adult heart function. Lastly, we summarized the mechanism of glucocorticoids in the regulation of muscle reprogramming and provided some perspectives on future directions.

## GLUCOCORTICOID SYNTHESIS AND METABOLISM IN THE FETUS

Regulation of fetal glucocorticoid synthesis and metabolism is a complex process, involving the HPA axis, the catalytic enzymes and the placenta. In humans, cortisol, one of the major forms of glucocorticoids peaks at 8–9 weeks of pregnancy, and then declines until undetectable at about 14 weeks of pregnancy (Parker et al., 1995; Goto et al., 2006). The serum cortisol levels stay low until the appearance of the surge in late gestation (Mesiano et al., 1993; Parker et al., 1995; Narasaka et al., 2001; Goto et al., 2006; Solano and Arck, 2020). The first peak of serum cortisol level is corresponding to the expression of the HSD3B2, an enzyme that is responsible for the critical timing of cortisol synthesis early in gestation. The surge of serum cortisol in late gestation is a coordination of increased HSD3B2 expression, elevated fetal cortisol production, and maternal cortisol crossing the placenta (Parker et al., 1995; Narasaka et al., 2001; Busada and Cidlowski, 2017). Cortisol peak appears at earlier stage of pregnancy which is important to promote the implantation of embryo and the decidualization of the uterine wall, as well as suppresses the maternal immune rejection to embryo (Busada and Cidlowski, 2017), whereas the surge at late gestation of fetal life is vital to the maturation of lung and many other organ systems which is crucial to survive for a life after birth (Solano et al., 2016; Busada and Cidlowski, 2017).

**FIGURE 1 |:**
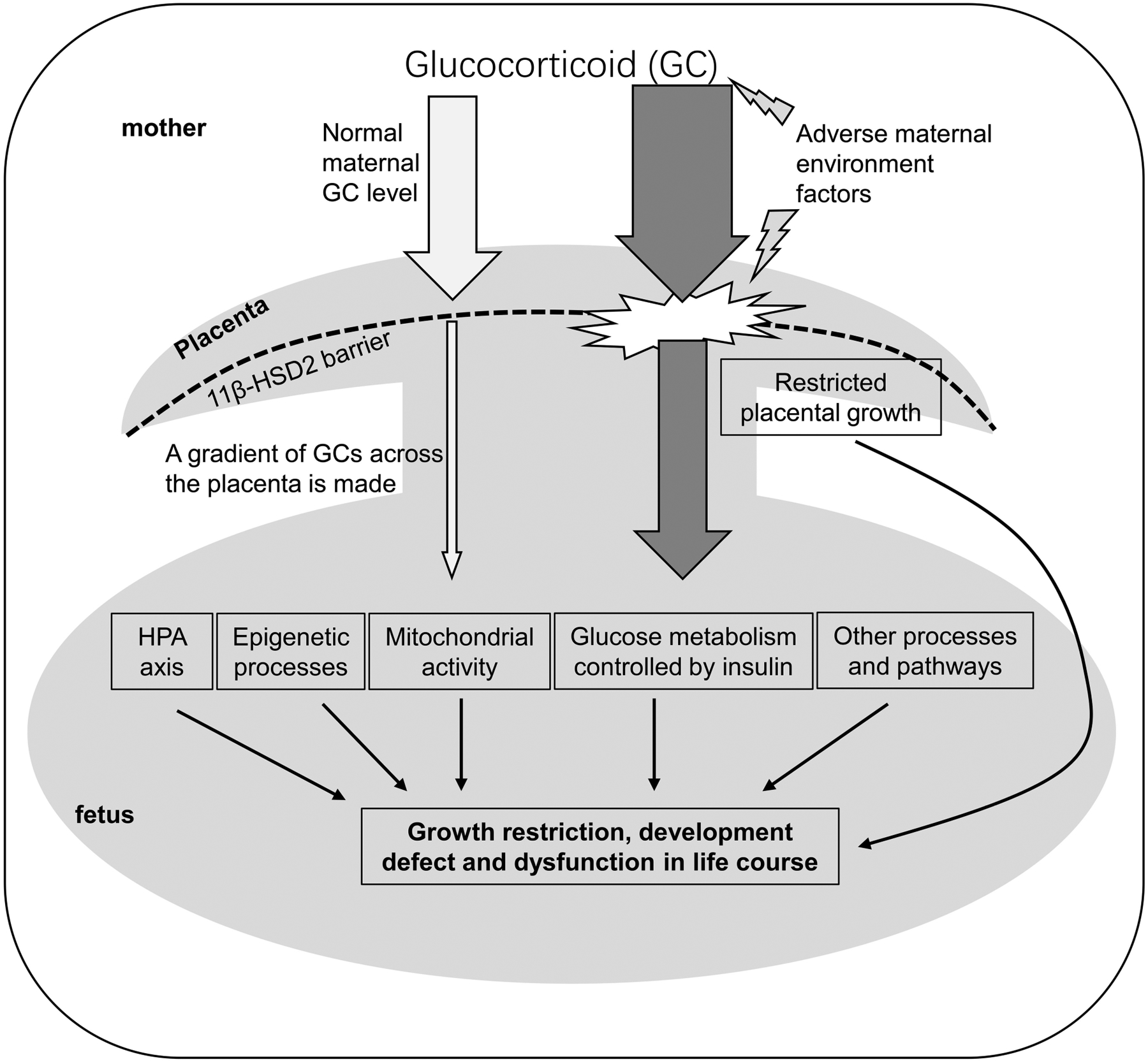
A schematic model to illustrate the mechanism that maternal glucocorticoids impact the programming of fetus. GC, Glucocorticoid; 11β-HSD2, 11β-hydroxysteroid dehydrogenase type2; HPA axis, hypothalamic-pituitary-adrenal axis.

In addition to glucocorticoid synthesis that determines the critical window of fetal glucocorticoid exposure, glucocorticoid metabolism controls gradients, or concentration of glucocorticoid in the fetus. Two enzymes 11β-hydroxysteroid dehydrogenase type 1 (11β-HSD1) and 2 (11β-HSD2) play a primary role in glucocorticoid metabolism and are highly expressed in the uterus, placenta and fetal tissues (Solano et al., 2016; Sand et al., 2019). The enzyme 11β-HSD1 primarily converts the inactive form of glucocorticoid 11-dehydrocorticosterone or cortisone to active form corticosterone in rodents or cortisol in human or livestock (Jamieson et al., 1995; Ricketts et al., 1998; Lamadé et al., 2021), while the 11β-HSD2 performs the opposite role as the 11β-HSD1 (Brown et al., 1993; McMullen et al., 2012; Chen et al., 2021). Interestingly, studies in mouse model showed that the 11β-HSD1 global depletion in dams did not influence fetal development, suggesting the role of 11β-HSD1 is not critical for normal fetal development (Kotelevtsev et al., 1997). However, studies in a sheep model with inhibition of 11β-HSD1 indicated that regulation of local cortisol concentration was essential for fetal development (Brooks et al., 2015). These contradictory studies in different animal models suggest that further investigation of the function of 11β-HSD1 is warranted in fetal development. In contrast, the enzyme 11β-HSD2 plays an essential role in controlling the mobilization of high gradient of maternal glucocorticoid into low concentration of serum glucocorticoid in the fetus (Krozowski et al., 1995; Meyer and Novak, 2021) (Figure 1). This gradient of glucocorticoid concentration from maternal to fetal serum allows the fetus not to be exposed to high maternal glucocorticoid level (McMullen et al., 2012). Studies showed that the defect of the 11β-HSD2 in mice usually resulted in the exposure of the fetuses to high level of corticosterone (another major form of glucocorticoids) *in utero* (Kotelevtsev et al., 1999), and also the chronic maternal stress such as poor maternal nutrition could facilitate maternal serum corticosterone to overcome the 11β-HSD2 barrier and elevate the fetal serum corticosterone level in a rat model (Bingham et al., 2013). The consequences of the elevated fetal corticosterone levels can lead to retarded fetal and placenta growth as well as reprogramming of the fetus to predispose to high risk of metabolic syndrome throughout the life in animal models and in humans (Ferrari et al., 1996; Bingham et al., 2013; Reynolds et al., 2013). In addition, the change of the activity of the 11β-HSD isoforms 1 and 2 in the placenta can also cause the abnormal exposure of maternal glucocorticoids and thereby lead to abnormal gene expression and altered patterns of growth and development (McMullen et al., 2012). For example, treatment of 11βHSD2 inhibitor carbenoxolone on pregnant dams in rats had the similar effect with maternal low protein diet; both treatments led to the reduced birth weight, and hypertension in offspring (Langley-Evans, 1997). The life-long trajectory of muscle growth and development could be one of the consequences regulated by antenatal glucocorticoids exposure (Jobe, 2020).

## EFFECTS OF GESTATIONAL GLUCOCORTICOID ON PRENATAL MUSCLE DEVELOPMENT AND POSTNATAL MUSCLE GROWTH

Skeletal muscle is formed during embryonic development. The formation of muscle cells or muscle fibers (also known as myogenesis) is a complex process and tightly regulated by myogenic regulatory factors (*Myf5, MyoD, Myogenin, Mrf4*), and many other genes (e.g., paired box transcription factors 3 (*Pax3*) and 7 (*Pax7*), *Meox1/2*, *Foxc1/2*) and signaling pathways (e.g., Wnt, FGF, IGF, HGF, BMP, Shh, Notch, p38 MAPK, NFAT) (detailed information can be referred to Bryson-Richardson and Currie, 2008; Eng et al., 2013; Chal and Pourquié, 2017; Asfour et al., 2018). There are two stages of myogenesis during prenatal muscle development, primary myogenesis, and secondary myogenesis. In livestock, for example, in swine fetuses, primary myogenesis occurs within about 38 days of gestation, and the secondary myogenesis takes place between 46 and 95 days of gestation (Wigmore and Stickland, 1983). During the primary myogenesis, primary muscle fibers are formed at early gestation stage which account for about 20% of total muscle fibers formed during prenatal myogenesis. Secondary muscle fibers are formed using primary muscle fibers as templates at fetal development stage. Secondary muscle fibers take up ~80% of total muscle fibers (Yan et al., 2013b). It is widely accepted that muscle fiber numbers are fixed after birth and postnatal muscle growth is mainly dependent on hypertrophy of existing muscle fibers (Yan et al., 2013b; Reynolds et al., 2019). In this regards, reduced muscle fiber number during prenatal muscle development will negatively impact postnatal muscle growth performance.

The critical time window that affects the number of muscle fibers is during the fetal developmental stage because the majority of muscle fibers are formed during this stage (Allen et al., 1999; Yan et al., 2013b). Numerous evidence have shown that environmental changes in uterus, particularly, poor maternal nutrition influences prenatal skeletal muscle development and postnatal muscle growth in different species because skeletal muscle has less priority for nutrient partitioning by comparing to other organs like brain, heart, liver, gut, and placenta (Zhu et al., 2006; Du et al., 2010). Thus, skeletal muscle development is especially vulnerable to nutrient availability (Reynolds et al., 2019). Studies in young and old sheep demonstrated that both maternal over- and under-nutrition resulted in reduced secondary muscle fiber numbers and an increase in the secondary to primary fiber ratio in late gestation fetal lambs as well as reduced muscle mass and muscle fiber cross-section area (Zhu et al., 2004, 2006; Fahey et al., 2005; Daniel et al., 2007; Huang et al., 2010; Yan et al., 2013a; Reed et al., 2014; Hoffman et al., 2016; Gauvin et al., 2020). Studies in pigs also showed that nutrition was a major factor for birthweight and muscle mass (Karunaratne et al., 2005; Jia et al., 2016). The larger offspring resulting from sufficient nutrition had more muscle fibers (Dwyer et al., 1994; Rehfeldt and Kuhn, 2006; Musser et al., 2007; Tilley et al., 2007). Meanwhile, studies in rodents and guinea-pigs also showed the similar effects of poor maternal nutrition on muscle fiber number from young offspring (Dwyer et al., 1995). Several comprehensive reviews have summarized consequences of maternal nutrition on skeletal muscle development which will not be detailed here (Brameld and Daniel, 2008; Du et al., 2010; Rehfeldt et al., 2011).

The mechanisms of poor maternal nutrition in fetal development have been extensively studied. Some studies in sheep reported that both maternal nutrition restriction and maternal over-nutrition during early- to mid-gestation elevated fetal and newborn plasma cortisol concentrations (Smith et al., 2018; Ghnenis et al., 2021). Although glucocorticoid is a well-known catabolic protein acting on skeletal muscle, only few studies reported the effect of glucocorticoid exposure on fetal muscle growth and development. One study using rats as model showed that *in utero* dexamethasone (a synthetic glucocorticoid) exposure reduced fetal skeletal muscle mass (Gokulakrishnan et al., 2012). In this study, the author concluded that fetal exposure to dexamethasone reduced fetal growth independent of its effects on maternal food intake, but maternal food intake was additive, while other reports in rats indicated that the retarded fetal growth due to exposure to dexamethasone administration was because of the secondary impact of decreased maternal food intake (Woods and Weeks, 2005; Woods, 2006). Later, a follow-up study from the same group in rats found that precocious exposure to dexamethasone *in utero* led to the relatively lower number of *Pax7*+ muscle progenitor cells but not distribution of these cells. The *Pax7* induces the expression of myogenic regulatory factor genes *Myf5* and *MyoD* (Olguín and Pisconti, 2012), and starts to express in dermomyotome of mature somites. The *Pax7*+ cells in mouse embryonic day 12.5 restrictedly differentiate to lineage of muscle cell (Lepper and Fan, 2010). After birth, *Pax7*+ cells can also be activated and fused themselves to adjacent myofiber to promote hypertrophy or regeneration (Chal and Pourquié, 2017). This study concluded that the effect of *in utero* dexamethasone exposure on fetal myonuclear accretion was independent of mild restriction of maternal food intake (Gokulakrishnan et al., 2017). These findings demonstrate how gestational glucocorticoid contributes to postnatal muscle growth because satellite cells are important to postnatal muscle fiber hypertrophy. A recent study in pregnant ewes infused with cortisol indicated that chronic increases in maternal cortisol concentrations, as in maternal stress, altered gene expression that is associated with mitochondrial function and metabolism in skeletal muscle (Joseph et al., 2020). They did not observe significant changes of insulin signaling which is a potential target in skeletal muscle of *in utero* glucocorticoids exposure (Jellyman et al., 2012), but significantly changed free radicals and cell apoptotic pathways (Joseph et al., 2020). Another study in sheep showed that the gene expression of the myosin heavy chain isoform IIX, was upregulated by cortisol infusion (Davies et al., 2021). This finding implies that gestational glucocorticoids may also affect the fiber type of fetal muscle in addition to fiber number (Gicquel et al., 2008). They also observed that in mitochondrial metabolism, mitochondrial content, biogenesis markers, substrate-specific respiration rates, abundance of electron transfer system complex I and adenine nucleotide translocator in skeletal muscle were increased in a muscle-specific manner when sheep fetuses were infused with cortisol during gestation (Davies et al., 2021). Although studies have been done extensively in adult muscle development and growth, few studies have been done in regard to the effects of gestational glucocorticoid on fetal muscle development. Therefore, further investigation will be guaranteed in the field in future.

## EFFECTS OF PRENATAL GLUCOCORTICOIDS ON HEART MUSCLE DEVELOPMENT AND ADULT HEART DISEASE

It is well-known that glucocorticoids are critical for the maturation of organs and tissues before birth and elevated glucocorticoid level at late gestation is essential to prepare for birth (Schwab et al., 2012; Rog-Zielinska et al., 2013a; Fowden et al., 2016). However, a handful of evidence have shown that excessive prenatal glucocorticoid exposure results in long-term adverse cardiovascular diseases (Fowden et al., 2016). Clinically, antenatal glucocorticoid therapy is commonly used in women who have the risk of preterm birth during pregnancy. Glucocorticoid treatment for preterm birth reduces respiratory distress syndrome, cerebral hemorrhage and necrotising enterocolitis as well as incidence of neonatal death (Fowden et al., 1998; Agnew et al., 2018; McGoldrick et al., 2020). Unfortunately, about half of women who had the antenatal glucocorticoid treatment did not go preterm, and conversely, they deliver babies at or near term (Razaz et al., 2015; Kemp et al., 2016; MakhiJaffet al., 2016; Grzeskowiak et al., 2018). This will cause potential exposure of babies to excessive synthetic glucocorticoids *in utero*, and thus result in potential short- or long-term adverse effects associated with cardiovascular function. Researches have been conducted to understand how excessive prenatal glucocorticoids (endogenous or exogenous) reprogram heart muscle and impact cardiovascular health during life course.

During normal development, the heart experiences extensive morphological and geometrical changes shortly before birth and continuously after birth through cardiomyocyte hyperplasia and hypertrophy in adaptation to increased mechanical and functional needs. Glucocorticoid is a ligand of GR, a nuclear receptor that recognizes and binds to the GREs of the targeted DNA (Oakley and Cidlowski, 2013). Activated GR in mouse fetal heart promoted cardiac morphological and geometrical changes (Rog-Zielinska et al., 2013a,b, 2015). The GR null mice developed immature and small heart that had both impaired systolic and diastolic function similar to the preterm heart (Rog-Zielinska et al., 2013a,b). Interestingly, another study with specific depletion of GR in only mouse heart and vascular smooth muscle showed that mice exhibited systolic dysfunction in late gestation with abnormal sarcomeric ultrastructure (Rog-Zielinska et al., 2013b) similar to null GR mice. However, GR specific depleted mice had a normal size heart, suggesting glucocorticoids regulate heart development and function in other different ways in addition to GR-regulated structural and functional changes. Researches also showed that excessive glucocorticoids due to antenatal synthetic glucocorticoid (dexamethasone) treatment altered feto-placental vasculature in human (Elfayomy and Almasry, 2014) which was supported by a subsequent study in the *Hsd11b2* knockout mice. These mice had antenatal glucocorticoid excess and intrauterine growth restriction (IUGR) and showed immature heart development and cardiac dysfunction in late gestation. Rescue of feto-placental vasculature restored cardiac function (Wyrwoll et al., 2016). Impact of glucocorticoids on fetal cardiomyocyte maturation has also been investigated with *in vitro* cultures. Mouse fetal cardiomyocytes treated with corticosterone or dexamethasone showed early maturation structurally and functionally (Rog-Zielinska et al., 2015). Studies in mice showed that Fetal heart development and maturation were impacted by mitochondrial metabolic capacity (Lai et al., 2008). Glucocorticoids can regulate fetal heart development and maturation through induction of PGC-1a, a key regulator for cardiac mitochondrial function through GR (Rog-Zielinska et al., 2015). Further, glucocorticoids can promote more active form of thyroid hormone T3 converted from T4 through inducing deiodinase 1 (D1) and D2 expression (Forhead and Fowden, 2014). Both glucocorticoids and thyroids are important hormones for fetal heart development and maturation by switching myofilament protein isoforms and increasing atrial natriuretic peptide (ANP) (Van Tuyl et al., 2004; Chattergoon et al., 2012). According to this mechanism, elevated glucocorticoid level at early gestation stage before the HPA axis starts to produce fetal thyroid hormones may have a compromised maturation of fetal organs by glucocorticoid along, which may impact more rodent fetal development than in humans or in sheep because thyroid hormones are synthesized earlier at mid-gestation in humans or sheep than in rodents at late gestation (Forhead and Fowden, 2014). This mechanism could be another important consideration for antenatal glucocorticoid therapy with a question about whether thyroid hormone should be administered with synthetic glucocorticoids or not. In addition, transient hyperoxia in neonatal rodent causes reduced cell number and increased cell hypertrophy which leads to a high risk for hypertrophic cardiomyopathy in adult life and vulnerability to pressure overload (Bertagnolli et al., 2014; Puente et al., 2014). Studies showed that glucocorticoids played a role in this process. Administration of dexamethasone to neonatal rats reduced cardiomyocyte number but increased cardiomyocyte hypertrophy (Gay et al., 2015).

The relationship between early and/or excessive exposure to glucocorticoid during pregnancy and impact of life course on cardiovascular disease is well-recognized. Also glucocorticoid is a widely accepted gatekeeper for the thrifty hypothesis of the fetal origins of diseases (Seckl and Holmes, 2007; Fowden et al., 2016). However, precise molecular and cellular mechanisms by which excessive glucocorticoid-induced cardiac remodeling and functional change in fetal development and its effects on cardiovascular disease later in life need be further explored. More information gained from the mechanistic studies will also help establish new antenatal glucocorticoid treatment protocol regarding optimal formulation, timing of dosage and efficacy at different gestational stages.

## POTENTIAL MECHANISMS OF GLUCOCORTICOIDS IN FETAL MUSCLE DEVELOPMENT

Fetal glucocorticoid level change occurs in several different ways. First of all, endogenous glucocorticoids need overcome the placental barrier through 11βHSD2 expressed on the placenta from the mother to the fetus (Chapman et al., 2013). However, studies showed that maternal stress in guinea pigs resulted in elevated level of cortisol in fetal serum, which subsequently caused fetal reprogramming including muscle formation and development (Dauprat et al., 1984) (Figure 1). Due to the low affinity of placental 11βHSD2 to synthetic glucocorticoid (sGC), sGC can readily cross the placenta to increase fetal serum glucocorticoid to have direct effect on fetal organs (McCabe et al., 2001). Secondly, glucocorticoids can play a direct role in regulating placental function (Figure 1). For example, both sGC and endogenous glucocorticoids promote expression and release of corticotropin-releasing hormone (CRH) from the placenta. Subsequently, CRH triggers both fetal and maternal HPA axis in humans (Torricelli et al., 2011). Further, this mechanism may differ for some species that do not produce CRH in placenta, which explains species differences in response to prenatal exposure to endogenous or exogenous glucocorticoids (Moisiadis and Matthews, 2014). The third action of glucocorticoids on placenta is placental growth restriction and altered placental vascularization and structure (Braun et al., 2013). These changes due to maternal stress alter fetal serum glucocorticoid level, and thus elicit many molecular and cellular processes related to cell growth and apoptosis, metabolism, inflammation, signal transduction, and transport (Wang et al., 2004) in fetal organ or tissue development including muscles. For example, one of the glucocorticoid targeted genes, tripartite motif containing 63 (*Trim63*), which encodes a E3 ubiquitin ligase muscle RING finger 1 (MuRF1), were upregulated in muscle atrophy (Waddell et al., 2008), implying that MuRF1 could be a mediator in fetal programming controlled by glucocorticoid.

Epigenetics is increasingly accepted as a potential mechanism of glucocorticoid action on fetal development through the regulation of gene expression (Moisiadis and Matthews, 2014).

Over the past decades, more and more evidence show that epigenetics regulates reprogramming of fetal organ systems such as cardiovascular system. Expression of a handful of genes (e.g., *Nr3c1/2*, *Crh*, *Pomc,* and *Hsd11b2*) in regulating HPA axis can be regulated through DNA methylation and histone modification by glucocorticoids (Newell-Price, 2003; Alikhani-Koopaei et al., 2004; Weaver et al., 2004; Mueller and Bale, 2008; De Filippis et al., 2013; Ferreira et al., 2021). DNA methylation is critical for vertebrate heart development and maturation through a number of processes such as gametogenesis and hematopoiesis (Patterson et al., 2010; Smith and Meissner, 2013; Gilsbach et al., 2014; Martinez et al., 2015). For an instance, DNA methylation inhibitor 5-AZA suppressed the regulation of dexamethasone in binucleation at day 4 postnatally and proliferation at day 7 postnatally, resulting in increased cardiomyocyte number at the heart of day 14 postnatally. This study suggested the relationship between glucocorticoids and DNA methylation in muscle cell proliferation and differentiation in a developing rat heart (Gay et al., 2015). Another study in rats found that maternal stress like maternal hypoxia reduced GR expression through DNA methylation and 5-AZA treatment reversed hypoxia-induced promotor methylation and restored GR expression (Xiong et al., 2016). Subsequent study from the same group found that DNA methylation bridged prenatal hypoxia and epigenetic regulation of GR expression in adult offspring in rats (Lv et al., 2019; Song et al., 2019b). In addition to DNA methylation, glucocorticoids can also regulate histone modifications and miRNAs. Increasing evidence show that miRNAs regulate generation of glucocorticoids in adrenal gland and in contrast, glucocorticoids also regulate cell survival, proliferation, and function partially through regulation of miRNA expression (Clayton et al., 2018). For example, in humans, miRNA-30c-5p and miRNA-125b-5p regulated expression of genes involved in cardiomyogenesis or cardiac function *via* glucocorticoid-mediated signaling pathway (Wang et al., 2014).

In addition to epigenetic regulation, glucocorticoids also play a role in the regulation of muscle metabolism. As an energy consuming tissue, striated muscle particularly requires the normal mitochondrial activity and glucose metabolism (Figure 1). The gestational glucocorticoids level in sheep was found to be closely correlated with the increase in mitochondrial oxidative phosphorylation capacity of skeletal muscle (Davies et al., 2020). Gestational exposure of glucocorticoids led to increased mitochondrial content, biogenesis markers, substrate-specific respiration rates, and abundance of electron transfer system complex I and adenine nucleotide translocator in a muscle-specific manner (Davies et al., 2021). Adverse maternal environment not only impacted the β-cell development and growth in fetal pancreas (Gicquel et al., 2008), also altered the response to insulin in other organs including cardiac and skeletal muscle through the mediation of glucocorticoids (Norris et al., 2011; Blanco et al., 2014; Ferreira et al., 2021). Muscle normally contributes around 75% of the post-prandial glucose utilization which depends on the embedding of glucose transporters 1 (GLUT1) and 4 (GLUT4) into membrane of myocytes in an insulin-sensitive manner (dos Santos et al., 2012; Blanco et al., 2014; Kondash et al., 2020). In both skeletal and cardiac muscle, expression of GLUT1 and GLUT4 was elevated after dexamethasone treatment *in utero* (Meyer and Zhang, 2007; Wyrwoll et al., 2008; Jellyman et al., 2012; Blanco et al., 2014), implying that the glucose metabolism of fetal muscle is impacted by glucocorticoids. However, another research in rats suggested that dexamethasone exposure indirectly restricted the glucose availability of fetus, because they found the glucose transport to the fetus had no significant change but glucose utilization of maternal tissues was competitively increased (Norris et al., 2011). Moreover, calcium-handling genes (Meyer and Zhang, 2007; Agnew et al., 2019), oxidative stress related genes (Joseph et al., 2020), the genes *Mstn* (Jia et al., 2016), *Pik3r1* (Kuo et al., 2012), *Trim63* (Waddell et al., 2008), *Bmp4*, *Tbx3*, *Acadm,* and *Nkx2-6* (Peng et al., 2018), were all found to be regulated by altered glucocorticoid level during fetal programming. Among them, gene *Pln* which encodes a protein regulates the activity of cardiac muscle sarcoplasmic reticulum Ca^2+^-ATPase (Kosmidis et al., 2015), *Pik3r1*, which mediates the glucose metabolism in response to insulin in myotubes (Kuo et al., 2012), and *Trim63* which is associated with skeletal muscle atrophy (Waddell et al., 2008) have been proved to contain glucocorticoid response elements. Some other genes, such as *Mstn* (Jia et al., 2016), Bmp4, *Tbx3*, *Acadm,* and *Nkx2-6* (Peng et al., 2018) are controlled by glucocorticoid through epigenetic modifications.

## PERSPECTIVES AND FUTURE DIRECTIONS

Numerous evidence reported in human and livestock as well as animal models suggested that glucocorticoids are key mediators and gatekeeper in fetal programming. The altered gestational glucocorticoid levels induced by adverse maternal environment reprogram the development, growth, and function of fetal skeletal and cardiac muscle through altered HPA axis. At the molecular and cellular level, glucocorticoid action is involved in a complex signaling network including epigenetic regulation, mitochondrial activity, glucose metabolism, cell cyclin, and differentiation. However, the molecular mechanism of the life-course or even trans-generational effect in myocytes controlled by aberrant glucocorticoid level is far from being fully understood. Which co-activators of glucocorticoid receptor participate in the regulation of fetal programming remains to be defined. Regulation of posttranscriptional process in fetal programming has not been well-studied yet. A lot of work still need to be done in future studies. Understanding of these fundamental questions would help develop intervention strategies to prevent adverse offspring outcomes. Targeting to glucocorticoids and their downstream molecules may provide specific intervention methods to improve farm animal production and performance as well as human health.
